# Management of pancreatic trauma in urban India: A multicenter study

**DOI:** 10.1016/j.amsu.2022.103564

**Published:** 2022-04-13

**Authors:** Devi Bavishi, Monty Khajanchi, Ramlal Prajapati, Anita Gadgil, Bhakti Sarang, Kapil Dev Soni, Amay Banker, Dhanashree Moghe, Martin Gerdin Wärnberg

**Affiliations:** a Seth GS Medical College and KEM Hospital, General Surgery, Mumbai, India; b WHO Collaboration Centre for Research in Surgical Care Delivery in Low and Middle-Income Countries, Mumbai, India; c BARC Hospital, General Surgery, Mumbai, India; d Terna Medical College and Hospital, General Surgery, Navi Mumbai, India; e JPN Apex Trauma Centre, AIIMS, Critical & Intensive Care, Delhi, India; f Department of Global Public Health, Karolinska Institutet, Stockholm, Sweden; g Function Perioperative Medicine and Intensive Care, Karolinska University Hospital, Solna, Sweden

**Keywords:** Abdominal injuries, Pancreas, Trauma

## Abstract

**Background:**

Pancreatic trauma occurs in 0.2–2% of patients with blunt trauma and 1–12% of patients with penetrating trauma. The mortality and morbidity rates range from 9 to 34% and 30–60% respectively. We aimed to review the management of pancreatic trauma in a multicenter database from India.

**Methods:**

We analyzed all patients who suffered a pancreatic injury and who were included in the multicenter prospective observational study ‘Towards Improved Trauma Care Outcomes (TITCO)’.

**Results:**

Of the 16047 trauma cases, 1134 (7.1%) patients suffered abdominal trauma. Of all those with abdominal trauma, 55 patients (4.9%) had injury to the pancreas. 28 patients (50.9%) with pancreatic trauma were managed conservatively. 27 patients (49.1%) underwent surgical exploration in the form of laparotomies. 11 procedures were undertaken for pancreas. A total of 45 (82%) patients had associated injuries along with pancreatic injury. Thorax (19) (including injuries to lung, pleura and ribs), liver (17), bowel (14) and spleen (13) were the most common associated injuries.

**Conclusion:**

Conservative management was as common as operative management in patients with pancreatic injuries. Most (80%) grade III/IV underwent operative treatment. Many patients (82%) had associated injuries.

**Level of evidence:**

III.

## Highlights

• Our study is a multicentric study from India to review management of pancreatic trauma.
• Most of the pancreatic injuries are associated with other intrabdominal injuries.

## Abbreviations

TITCO: Towards Improved Trauma Care Outcomes
KEM: King Edward Memorial Hospital
LTMGH: Lokmanya Tilak Municipal General Hospital
JPNATC: Jai Prakash Narayan Apex Trauma Centre
SSKM: Seth Sukhlal Karnani Memorial Hospital
ISS: Injury Severity Score
ICD –: International Classification of Diseases
OIS: Organ Injury Scale
AAST: American Association for the Surgery of Trauma
CT: Computed Tomography
SBP –: Systolic Blood Pressure
HR –: Heart Rate
RR –: Respiratory Rate
OM –: Operative Management
FAST: Focused Assessment with Sonography for Trauma
EAST: Eastern Association for the Surgery of Trauma
WTA: Western Trauma Association
WSES: World Society of Emergency Surgery
MRI –: Magnetic Resonance Imaging
ERCP: Endoscopic Retrograde Cholangiopancreatography

## 1 Introduction

In patients with polytrauma, abdominal trauma is common with an incidence of 7–10% [1,2]. The incidence of pancreatic injury in abdominal trauma is 3–12% [3]. In a population-based study of 52,000 trauma patients in Scotland, the incidence of pancreatic injuries was 0.2% [4]. Pancreatic trauma occurs in 0.2–2% of patients with blunt trauma and 1–12% of patients with penetrating trauma [5]. The mortality and morbidity rates range from 9 to 34% and 30–60% respectively [6].

The retroperitoneal location of pancreas partly explains the low incidence of pancreatic trauma in abdominal injuries. Its location also means that pancreatic injury is a marker of severe trauma. The pancreas’ association with surrounding visceral and vascular injuries may lead to concomitant injuries and significant morbidity - including pancreatic fistula, pseudocyst, pancreatitis, repeated surgeries [7], and mortality [8].

The overall management depends on the associated injuries and the status of the main pancreatic duct [6]. There are single centre studies [[9], [10], [11], [12], [13], [14]] from India and China that highlight the demographics and management of pancreatic trauma patients, but no multicenter studies from low-middle income countries [4]. We therefore aimed to review the management of pancreatic trauma in a multicenter database from India.

## 2 Materials and methods

### 2.1 Registration

In accordance with the Declaration of Helsinki, our study has been registered with OSF registry retrospectively. The registration DOI is 10.17605/OSF.IO/M4QWF.

### 2.2 Ethical approval

The TITCO project was granted waivers of informed consent from all study centers. The study received approval from the institutional ethics committee of the four centers involved in the study. The ethics approval registration numbers were EC/NP-279/2013 RP-O1/2013 from the All India Institute of Medical Sciences Ethics Committee, IEC/11/13 from the Lokmanya Tilak Municipal Medical College and Lokmanya Tilak Municipal General Hospital Institutional Ethics Committee, IEC/279 from the Institute of Post Graduate Medical Education and Research (IPGME&R) Research Oversight Committee (Institutional Ethics Committee), and IEC(I)OUT/222/14 from the Seth GS Medical College and King Edward Memorial Hospital Institutional Ethics Committee.

### 2.3 Study design

We analyzed all patients who suffered a pancreatic injury and who were included in the multicenter prospective observational study ‘Towards Improved Trauma Care Outcomes (TITCO)’ in India conducted from October 2013 to December 2015 [15].

### 2.4 Setting

The study was conducted in four public university hospitals in India. These urban referral tertiary care hospitals are situated in Kolkata, Mumbai (2-centers) and Delhi, cities with populations of more than 10 million. The hospitals were King Edward Memorial Hospital (KEMH) and Lokmanya Tilak Municipal General Hospital (LTMGH) in Mumbai, Jai Prakash Narayan Apex Trauma Centre (JPNATC) in New Delhi and the Institute of Post-Graduate Medical Education and Research and Seth Sukhlal Karnani Memorial Hospital (SSKM) in Kolkata. Each of these hospitals receive around 20 to 30 major trauma patients per week. They have 24-h emergency services, imaging, operating theatres, and sub-specialty available.

### 2.5 Data collection

One dedicated project officer per hospital collected the data. They collected data by directly observing care for patients admitted during their shifts and by extracting data from hospital records for patients admitted outside their shifts. Patients were followed up until discharge or death, whichever occurred first over a 30-day period. Certified coders calculated the Injury Severity Score (ISS) of each patient based on injury descriptors extracted from patient records, including imaging reports and surgical notes.

### 2.6 Participants

TITCO included all patients with life or limb threatening injuries admitted to the participating hospitals. For this analysis, patients with pancreatic injury corresponding to the International Classification of Diseases (ICD) 10 code S36.2 were extracted from the TITCO database.

### 2.7 Variables

Demographic variables analyzed were age, sex, mechanism of injury, mode of transport, and type of injury (blunt or penetrating). Clinical profile was analyzed in terms of vital signs, imaging, intervention, length of stay and outcome (death/discharge). Pancreatic injuries were graded according to the Organ Injury Scale (OIS) developed by the American Association for the Surgery of Trauma (AAST) from Computed Tomography (CT) findings and operative data [17]. Data were not sufficient to differentiate grade I from II, hence they were combined as I/II. Data were not available for 4 patients and 17 patients could not be classified due to incomplete records and were coded as data not available. Length of stay and in-hospital mortality were entered.

### 2.8 Analyses

Means with standard deviations were calculated for continuous variables, and medians with interquartile range were calculated for non-normal continuous variables. Categorical variables are presented as counts and proportions.

Our work has been reported in line with the STROCSS criteria [16].

## 3 Results

Of the 16047 trauma cases, 1134 (7.1%) patients suffered abdominal trauma. Of all those with abdominal trauma 55 patients (4.9%) had injury to the pancreas. The mean age of these 55 patients with pancreatic injury was 27(SD = 13) years, 50 (91%) were males, and 51 (93%) suffered blunt trauma. The main mechanism of injury was road traffic injury (32, 58%) followed by falls (14, 25.5%). More than two thirds (37, 67%) of the patients were transferred to the participating centers. Most came to the participating centers via ambulance (38, 69%). Out of the 55 patients 13 died in hospital within 30 days (24%). (Table 1).

**Table 1:** Demographics, Physiology and Outcomes of patients with Pancreatic trauma.

Variables	Patients n = 55	Missing values (n)
Age in years, mean (SD)	27 (13)	0
Male sex, n (%)	50 (90.9)	0
Mode of injury, n (%)		0
- Blunt	51 (92.7)	
- Penetrating	4 (7.3)	
Mechanism of injury, n (%)		0
- Road traffic injury	32 (58.2)	
- Fall	14 (25.5)	
- Railway	2 (3.6)	
- Assault	4 (7.3)	
- Other	3 (5.5)	
Transferred, n (%)	37 (67)	0
Mode of transport, n (%)		0
- Ambulance	38 (69)	
- Police	4 (7)	
- Private car	9 (16)	
- Taxi	4 (7)	
SBP in mmHg, median (IQR)	120 (110–128)	3
HR as beats per min, median (IQR)	88 (80–110)	0
RR per min, median (IQR)	18 (16–20)	3
Hemoglobin in g/dl, median (IQR)	12.5 (11.8–13.3)	6
ISS score, mean (SD)	15.2 (8)	12
- Mild (<9), n (%)	8 (14.6)	
- Moderate (9–15), n (%)	14 (25.4)	
- Severe (16–25), n (%)	16 (29.1)	
- Profound (>25), n (%)	5 (9.1)	
- Missing, n (%)	12 (21.8)	
Mortality, n (%)	13 (23.6)	

SBP – systolic blood pressure, HR – heart rate, RR – respiratory rate, ISS – Injury severity score. Continuous variables are represented by median in parentheses by IQR.

Categorical variables are represented as counts and in parenthesis as proportions.

**Fig. 1:**
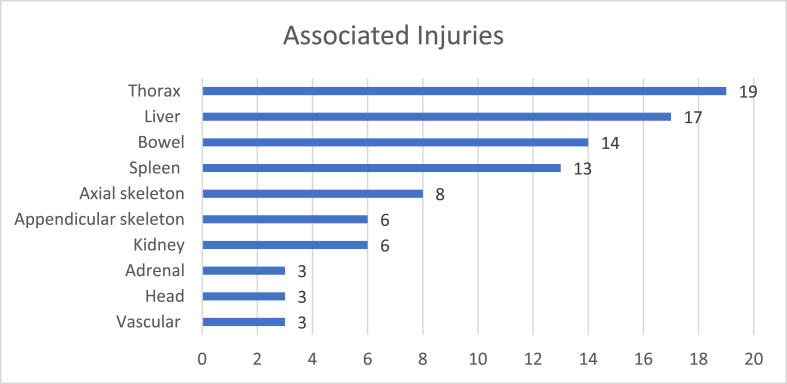
Associated other organ injuries with pancreatic trauma.

A total of 45 (82%) patients had associated injuries along with pancreatic injury. Thorax (including injuries to lung, pleura, and ribs), liver, spleen and bowel were the most common associated injuries (Fig. 1).

### 3.1 Diagnostic modalities

Out of 55 patients, Focused Assessment with Sonography for Trauma (FAST) was done in 52 patients (94.5%). Out of three patients in whom FAST was not done two underwent exploratory laparotomy immediately. A total of 47 (85%) patients underwent CT imaging. Four (7%) patients underwent exploratory laparotomy without the CT scan. The remaining four patients did not undergo CT nor laparotomy and their details and reasons were not available.

### 3.2 Pancreatic trauma grading

19 patients (34.5%) had grade I/II pancreatic injury, 10 patients had grade III injury, four patients had grade IV injury and only one patient had grade V pancreatic injury (Table 2).

**Table 2:** Management of pancreatic injuries according to grade (OM-operative management).

	Conservative	Operative				Combined	Length of stay (days in median)
Grade of pancreatic trauma		Laparotomies = a+b + c	OM for pancreas ± other intra-abdominal organ (a)	OM for intra-abdominal organ other than pancreas (b)	OM for unspecified reason (c)	Total (%)	
I/II	13	6	2	3	1	19 (34.6)	13
III	1	9	6	0	3	10 (18.2)	12
IV	2	2	1	0	1	4 (7.3)	16
V	0	1	0	1	0	1 (1.8)	17
NA	12	9	2	4	3	21 (38.2)	12
Total	28	27	11	8	8	55	13

### 3.3 Conservative management

28 patients (28,50.9%) with pancreatic trauma were managed conservatively. 13 patients (13,23.6%) had grade I/II pancreatic injury, one patient had grade III injury and two patients had grade IV injury. Of the patients managed conservatively, six patients (20%) died, two with grade I/II injury, one with grade IV injury and three who could not be assigned a grade. The median length of stay for patients managed conservatively was 13 days.

### 3.4 Operative management

27 patients (49.1%) underwent surgical exploration in the form of laparotomies. 11 procedures were undertaken for pancreatic injury, which ranged from one pancreatic laceration repair for a grade I/II injury, nine distal pancreatectomies were done of which one was for grade I/II pancreatic injury and six were for grade III pancreatic injuries. Grades of two other distal pancreatectomy were not available. One pancreaticoduodenectomy was done for grade IV injury.

**Fig. 2:**
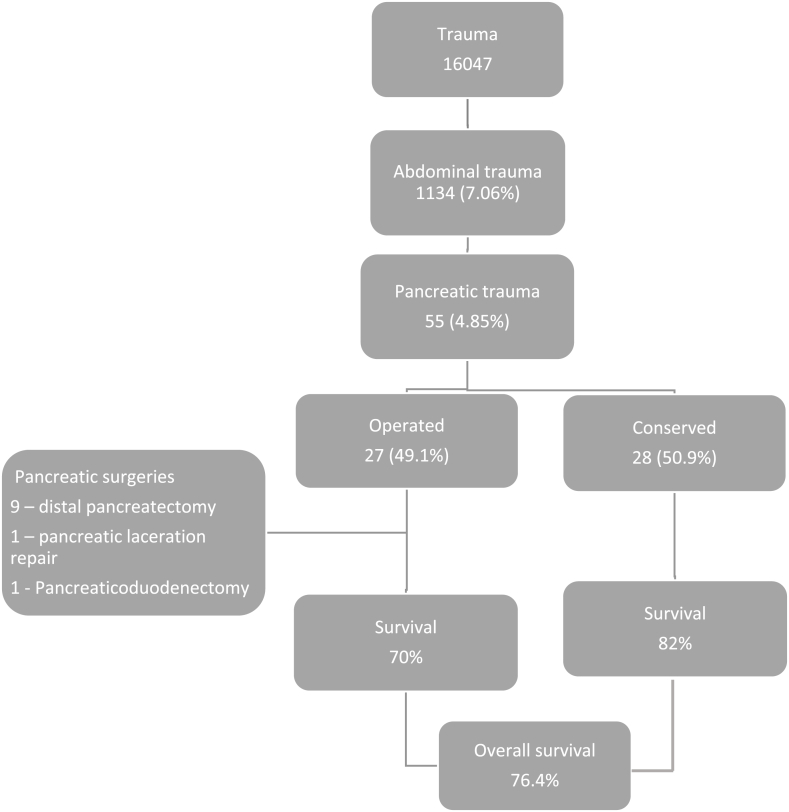
Patient flow.

Of the six operated grade I/II patients, three patients were operated for right liver laceration, jejunal perforation and colonic transection. Two patients were operated for pancreatic injuries, one operated for pancreatic laceration where repair was performed with management of grade IV splenic injury, and the other was with pancreatic contusion in whom a distal pancreatectomy was done. All patients with grade III [9] pancreatic injury were operated, except one pediatric age group patient. Details of the other operated patient with grade IV pancreatic injury was not available. (Fig. 2).

Two patients with grade IV injuries were conserved. One with no associated injuries, while the other with liver injuries and lesser sac hematoma, both who were young adults. More details about the reason for the conservative management of these patients could not be inferred from the data. The only patient with grade V injury in our study was operated for transection at D2 segment of duodenum, probably along with some surgery for pancreas, details of which are not available.

Of the patients managed operatively, seven patients died (28%), one with grade I/II injury and one with grade IV injury and five could not be graded. The patient with grade I/II injury died due to head injury in one day, the patient had grade IV injury who died after 15 days of hospital stay, most likely due to pancreatic injury, as there were no other injuries and one patient with grade V injury died due to pancreatico-duodenal injuries after 18 days of hospital stay. All operated patients with grade III and IV injury survived.

Out of 25 patients who were operated, surgery lasted for 1–3 h in 23 patients, > 4 h in one patient and data was not available for one patient. The median length of stay was 12 days (IQR 8–27 days), for patients who survived as well as those who died.

## 4 Discussion

We found that conservative management was more common in low grade injuries compared with high-grade injuries, which were operated on. We also found that 82% of patients had at least one injury other than pancreas.

Out of 19 patients with grade I/II injuries in our study, 13 were managed conservatively, and six patients needed surgery. The Eastern Association for the Surgery of Trauma (EAST) guidelines, Western Trauma Association (WTA) algorithm and World Society of Emergency Surgery (WSES) guidelines for management of pancreatic injuries recommends conservative management of AAST grade I and II (low-grade) blunt pancreatic injury [[18], [19], [20]]. Operative management in low grade pancreatic injuries is usually done for associated injuries [4]. Pancreatic injuries are often incidentally detected intraoperatively while a laparotomy is done for other injuries. At that time, surgical hemostasis and drainage is recommended. Resection is avoided due to delayed complications like formation of pseudocyst and fistulae [18,20]. In our cohort, three patients were operated for associated injuries and two patients were operated due to pancreatic injuries. In our experience, intra-operatively, it may not be possible to detect a pancreatic ductal injury and sometimes a surgeon may go ahead with a distal pancreatectomy if there are major bruises over the pancreatic tail and clinical suspicion of pancreatic ductal injury in the tail even though a CT scan did not find it.

Out of 10 patients with grade III injuries, nine were operated and six underwent surgery for pancreatic injury. All six underwent distal pancreatectomy with or without splenectomy. Distal pancreatectomy is recommended for grade III injuries [18,21]. However, in select hemodynamically stable patients, with proximal pancreatic body injuries, conservative management with endoscopic and percutaneous interventions increases success of non-operative management [19]. Distal injuries should undergo operative management in the form of distal pancreatectomy [19].

Only one patient of grade III injury was conserved, probably because he was a child with better chances of recovery with conservative management and hence lesser morbidity. In the pediatric population, there have been studies demonstrating success of non-operative management in management of high-grade pancreatic injuries [7]. For pediatric pancreatic trauma, there exist no standard guidelines or recommendations for management [22,23]. A systematic review recommends conservative management of low-grade pancreatic injuries (grade I-II). However, there is no consensus on the recommendation for high grade injury (grade III IV V) [22].

Of the four patients with grade IV injuries, two were operated and two were conserved. EAST guidelines recommend operative management of high-grade blunt pancreatic injury [18]. WSES guidelines have similar recommendations for high grade pancreatic trauma; operative management in the form of debridement, over sewing the proximal pancreatic stump, distal drainage with pancreatico-jejunostomy and sometimes a staged pancreaticoduodenectomy. If a patient is hemodynamically stable, has no other associated injuries needing surgery, has availability of other conservative interventions at high resource centers, non-operative management can be tried [19]. There are recent trends of conservative management even for high grade injuries as studies have failed to show a significant difference in mortality outcome with resection over conservative management, only a decrease in length of stay [24].

Of the 28 patients (50.9%) managed conservatively, six patients (20%) died. Out of these patients, two patients had grade I/II injuries and they mostly succumbed to other associated high-grade injuries. Out of the 27 patients (49.1%) who underwent surgical exploration in the form of laparotomies. eight patients died (30%). Mortality due to pancreatic trauma is difficult to ascertain because of multiple associated injuries in patients especially due to road traffic injuries leading to blunt multi-system trauma.

Pancreatic injury most commonly occurs with other associated injuries in as much as 90% of patients [25,26]. In our study 45 patients (82%) had associated injuries. The liver, spleen, stomach, duodenum, and colon are the most injured organs. A study noted associated injury to the liver (26%), colon or small bowel (25%), major vessels (25%), duodenum (24%), stomach (19%), spleen (12%) and kidney (10%) [27] which is like our study where liver is the second most common injury in 31% patients and spleen in 24% patients, suggesting that larger solid organ and organs with close proximity to pancreas are more prone to injuries with pancreas. 19 (35%) patients had thoracic injuries. Although thoracic injuries are not widely reported and only one study reported thoracic injuries in 53% patients with blunt trauma [28], in our study it was the most common associated injury. This finding could be due to the overwhelming predominance of blunt trauma in our study. Contrary to other studies with a high number of concomitant vascular injuries in other studies [2], our study had only 3 patients with vascular injury. This may be due to the high volume of low impact blunt trauma in our setup leading to less shear force needed for vascular injury.

### 4.1 Limitations

Firstly, data regarding the Magnetic Resonance Imaging (MRI), Endoscopic Retrograde Cholangiopancreatography (ERCP) and interventional radiology done in our patients were not available. Second, the profile and outcomes in pancreatic injury reported by us are from tertiary care university hospitals and cannot be generalized to smaller centers and to hospitals in the villages where the resources available are limited and variable.

## 5 Conclusion

Conservative management was as common as operative management in patients with pancreatic injuries. Most (80%) grade III/IV underwent operative treatment. 45 patients (82%) had associated injuries.

## Ethics approval

The TITCO project was granted waivers of informed consent from all study centers. The study received approval from the institutional ethics committee of the four centers involved in the study. The ethics approval registration numbers were EC/NP-279/2013 RP-O1/2013 from the All India Institute of Medical Sciences Ethics Committee, IEC/11/13 from the Lokmanya Tilak Municipal Medical College and Lokmanya Tilak Municipal General Hospital Institutional Ethics Committee, IEC/279 from the Institute of Post Graduate Medical Education and Research (IPGME&R) Research Oversight Committee (Institutional Ethics Committee), and IEC(I)OUT/222/14 from the Seth GS Medical College and King Edward Memorial Hospital Institutional Ethics Committee.

## Availability of data and materials

This is a subgroup analysis of patients with pancreatic injury from a prospective trauma registry study named ‘Towards Improved Trauma Care Outcomes’ (TITCO) in India. [Roy N, Gerdin M, Ghosh S et al. (2016) 30-day in-hospital trauma mortality in four urban university hospitals using an Indian Trauma Registry. World J Surg 40:1299–1307]. Data can be requested from the authors.

## Funding

The TITCO funding agencies were the Swedish 10.13039/501100005349National Board of Health and Welfare and the 10.13039/501100004102Laerdal Foundation. The authors declare that these agencies had no influence on the conceptualization or findings of this study.

## Statement on ethical standard

All procedures performed were in accordance with the ethical standards of the institutional and national research committee and with the 1964 Helsinki Declaration and its later amendments.

## Consensus and publication statement

This is a subgroup analysis of patients with pancreatic injury from a prospective trauma registry study named ‘Towards Improved Trauma Care Outcomes’ (TITCO) in India. [Roy N, Gerdin M, Ghosh S et al. (2016) 30-day in-hospital trauma mortality in four urban university hospitals using an Indian Trauma Registry. World J Surg 40:1299–1307].

This analysis and manuscript has not been published and is neither under consideration for publication elsewhere.

## Author contributions

MK came up with the idea. DB, MK worked on literature search, design. DB, MK, RP worked on data analysis and interpretation. DB, MK, RP, AG, BS, KDS, AB, DM, MGW, worked on manuscript preparation. DB, MK, RP, AG, BS, KDS, AB, DM, MGW edited the manuscript. All authors reviewed the manuscript. DB submitted the final manuscript.

## Consent

The TITCO project was granted waivers of informed consent from all study centers.

## Registration of research studies

1. Name of the registry: Open Science Framework.

2. Unique Identifying number or registration ID: m4qwf, Registration DOI: 10.17605/OSF.IO/M4QWF.

3. Hyperlink to your specific registration (must be publicly accessible and will be checked): https://osf.io/m4qwf.

## Guarantor

Martin Gerdin Wärnberg, MD, PhD.

Address.

1. Department of Global Public Health, Karolinska Institutet, Stockholm, Sweden

2. Function Perioperative Medicine and Intensive Care, Karolinska University Hospital, Solna, Sweden.

Email: martin.gerdin@ki.se.

Phone number: +46 70 853 95 98.

## Provenance and peer review

Not commissioned, externally peer reviewed.

## Declaration of competing interest

All authors declare that they have no competing interest to disclose.
